# High body mass index in rheumatoid arthritis: why we should promote physical activity

**DOI:** 10.1186/s13075-016-1209-5

**Published:** 2017-01-10

**Authors:** M. Hugo, N. Mehsen-Cetre, A. Pierreisnard, E. Pupier, B. Cherifi, T. Schaeverbeke, V. Rigalleau

**Affiliations:** Endocrinologie-Nutrition, CHU de Bordeaux, USN, Hôpital Haut-Lévêque, Avenue de Magellan, 33600 Pessac, France

We were interested in the recent publication of Albrecht et al. on body mass index (BMI) distribution in rheumatoid arthritis (RA) [1]. According to this analysis of three large cohorts, the majority of patients with RA are overweight [1]. Low remission rates and common metabolic syndrome indicate that the increasing BMI in RA should be treated, but weight loss may not be the solution as it has been linked to increased mortality [2]. Working on nutritional status in RA, we had the opportunity to compare the body composition analysis (DEXA) and physical activity levels recorded over 3 days with Actimeters (SenseWear Arm Bands, Body Media, Stanford, CA, USA) in overweight versus normal-weight patients. We feel that our results may help orientate management despite the obesity paradox in RA.

As depicted in Table 1, the main characteristics of the patients (age and gender) and their disease (duration, DAS28-ESR, and use of corticosteroids) were similar in the overweight and normal subjects. The rates of rheumatoid cachexia and osteopenia were dramatically reduced in overweight patients. Over the whole group, BMI were positively related to bone mass (*r* = +0.29, *p* < 0.05) and the rachis T scores (*r* = +0.36, *p* < 0.01). The overweight patients had lower levels of physical activity, and BMI was negatively related to these levels: metabolic equivalent tasks (METs) (*r* = –0.60, *p* < 0.001) and daily duration of physical activity (*r* = –0.41, *p* < 0.005).

**Table 1:** Characteristics of patients with rheumatoid arthritis and who were overweight or obese (*N* = 27) as compared to patients of normal weight (*N* = 29)

	Normal weight	Overweight or obese	*p*
Gender (% men)	20%	21%	NS
Age (years)	56 ± 12	58 ± 9	NS
Duration of rheumatoid arthritis (years)	6.9 ± 8.3	7.2 ± 6.6	NS
DAS28-ESR	3.7 ± 1.7	4.1 ± 2.0	NS
Treated by corticosteroids (%)	65%	53%	NS
Nutritional status			
Body mass index (kg/m^2^)	22.1 ± 2.2	30.5 ± 6.9	<0.0001
Fat (%)	32.1 ± 10.2	38.0 ± 8.2	<0.05
Fat-free mass index (kg/m^2^)	15.2 ± 1.7	18.6 ± 2.7	<0.001
Metabolic syndrome (%)	10.3%	39.3%	<0.05
Rheumatoid cachexia (%)	34.5%	3.7%	<0.01
Bone status			
Bone mass (g)	1978 ± 365	2223 ± 398	<0.05
Rachis T score	–1.1 ± 1.3	0.0 ± 1.4	<0.005
Osteopenic, rachis (%)	66%	24%	<0.005
Femoral neck, T score	–1.3 ± 1.2	–0.6 ± 1.2	<0.05
Osteopenic, femoral (%)	74%	40%	<0.05
Actimetry			
Metabolic equivalent tasks	1.52 ± 0.32	1.24 ± 0.25	<0.005
Duration of physical activity (min/day)	109 ± 99	59 ± 71	<0.05

*DAS28-ESR* Disease Activity Score in 28 joints-erythrocyte sedimentation rate, *NS* not significant

The body composition analysis of our overweight patients shows that some of their nutritional characteristics should be preserved by therapeutic intervention: less rheumatoid cachexia, that is known to reduce life expectancy, and less osteopenia, whereas the risk of fractures is doubled in RA [3]. The reduced levels of physical activity in overweight RA patients has been reported using questionnaires [4], but to our knowledge this has not yet been demonstrated with more objective actimetry measurements as we have performed. Improving these low levels of activity should be beneficial for the metabolic syndrome of overweight patients. Exercise is also considered beneficial for osteoporosis and for rheumatoid cachexia. The main limitation of interventions on physical activity is their modest results in terms of weight loss [5], while mortality may be increased by frank and unintentional weight loss in RA [2].

## Abbreviations

BMI: Body mass index
MET: Metabolic equivalent task
RA: Rheumatoid arthritis
